# Glucosinolates and Omega-3 Fatty Acids from Mustard Seeds: Phytochemistry and Pharmacology

**DOI:** 10.3390/plants11172290

**Published:** 2022-09-01

**Authors:** Gitishree Das, Ourlad Alzeus G. Tantengco, Rosa Tundis, Joyce Ann H. Robles, Monica Rosa Loizzo, Han Seung Shin, Jayanta Kumar Patra

**Affiliations:** 1 Research Institute of Integrative Life Sciences, Dongguk University-Seoul, Goyang-si 10326, Korea; 2 College of Medicine, University of the Philippines Manila, Manila 1000, Philippines; 3 Department of Pharmacy, Health and Nutritional Sciences, University of Calabria, 87036 Rende, Italy; 4 Department of Food Science & Biotechnology, Dongguk University-Seoul, Goyang-si 10326, Korea

**Keywords:** *Brassica* spp., pharmaceutical properties, *Sinapsis* spp., glucosinolates, omega-3 fatty acids

## Abstract

Seeds from mustard (genera *Brassica* spp. and *Sinapsis* spp.), are known as a rich source of glucosinolates and omega-3 fatty acids. These compounds are widely known for their health benefits that include reducing inflammation and lowering the risk of cardiovascular diseases and cancer. This review presented a synthesis of published literature from Google Scholar, PubMed, Scopus, Sci Finder, and Web of Science regarding the different glucosinolates and omega-3 fatty acids isolated from mustard seeds. We presented an overview of extraction, isolation, purification, and structure elucidation of glucosinolates from the seeds of mustard plants. Moreover, we presented a compilation of in vitro, in vivo, and clinical studies showing the potential health benefits of glucosinolates and omega-3 fatty acids. Previous studies showed that glucosinolates have antimicrobial, antipain, and anticancer properties while omega-3 fatty acids are useful for their pharmacologic effects against sleep disorders, anxiety, cerebrovascular disease, neurodegenerative disease, hypercholesterolemia, and diabetes. Further studies are needed to investigate other naturally occurring glucosinolates and omega-3 fatty acids, improve and standardize the extraction and isolation methods from mustard seeds, and obtain more clinical evidence on the pharmacological applications of glucosinolates and omega-3 fatty acids from mustard seeds.

## 1. Introduction

Mustard belongs to the family Brassicaceae and is valued for its spicy and pungent dried seeds. Some of the well-known species of mustard include black mustard, *Brassica nigra* (L.) W. D. J. Koch, brown mustard, *Brassica juncea* (L.) Czerniak, *Brassica rugosa* Hort., *Sinapis juncea* L., white mustard, and *Brassica hirta* Moench [1]. The mustards grow best in sandy loam soils with limited rainfall. It is usually cultivated under temperate climates, but it can also be grown in tropical and subtropical regions. It is considered as one of the first domesticated crops and is commonly grown in Asia, North Africa, and Europe [2]. Mustard plants are commonly used in the food industry. White mustard is commonly used as food flavoring while black and brown mustards are generally used for their aroma. Some mustard plants such as *B. alba* and *B. juncea* are also used by traditional healers as herbal medicine to treat arthritis, colds, cough, sore throat, muscle pain, and diabetes [1].

**Figure 1:**
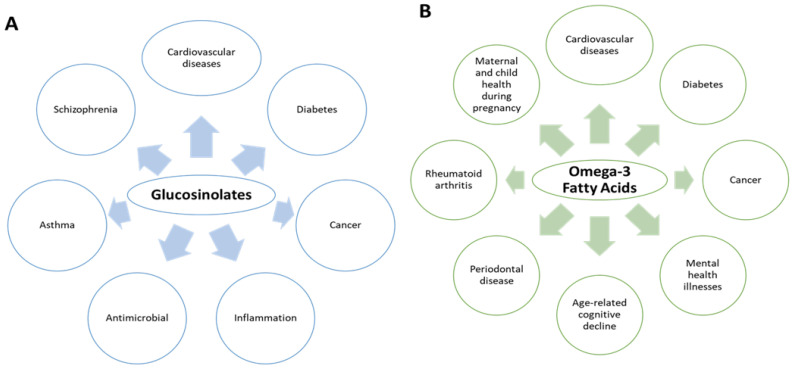
Known human health benefits of glucosinolates (**A**) and omega-3 fatty acids (**B**).

Mustard seeds contain several bioactive compounds which include glucosinolates (GSLs) and omega-3 fatty acids [3]. GSLs are composed of three compartments: β-thioglucose, thiohydroximate-O-sulfonate, and a variable aglycone side chain derived from an α-amino acid [4]. On the other hand, omega-3 fatty acids are PUFAs that contain more than one carbon–carbon double bond in their backbone. They are widely known for their health benefits that include reducing inflammation and lowering the risk of heart diseases and cancer [5]. Additionally, consumption of glucosinolates by humans causes a positive effect on the body and have anticarcinogenic properties including contribution to the bioactive nature of the oil obtained from the mustard seeds [6]. Although these two compounds are unrelated, their presence in the mustard seeds and oils is beneficial to humans. Figure 1 summarizes the known health benefits of these compounds in humans [7,8,9,10]. This paper is an extensive review of the different GSLs and omega-3 fatty acids that can be found in mustard seeds. The review discusses the major extraction procedures to isolate these compounds and the different pharmacological applications along with the mechanism of action. Lastly, ongoing clinical studies using GSLs and omega-3 fatty acids from mustard seeds are also described in this review. All collected data have been obtained from different databases such as Google Scholar, PubMed, Scopus, Sci Finder, and Web of Science.

## 2. Major Bioactive Compounds in Mustard Seeds: Glucosinolates and Omega-3 Fatty Acids

**Figure 2:**
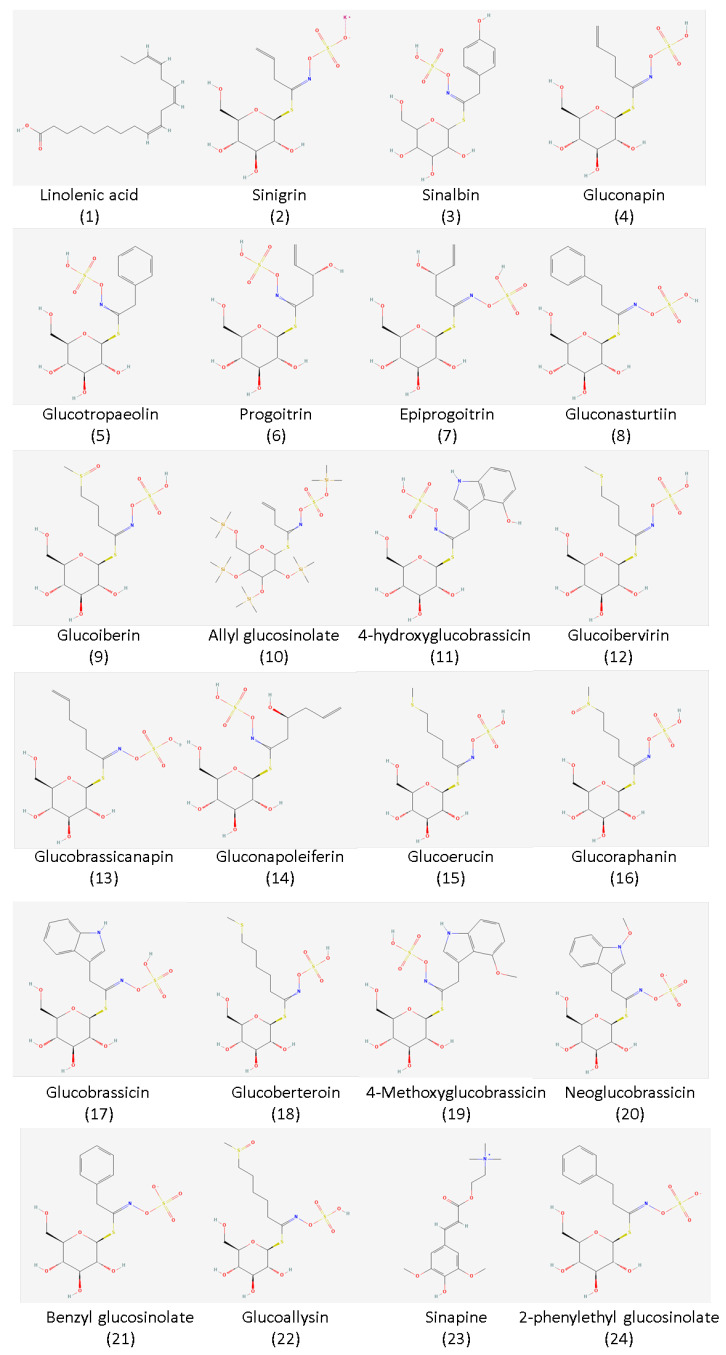
Chemical structure of the most common GSLs isolated from the seeds of mustard plants. The chemical structures were obtained from PubChem (https://pubchem.ncbi.nlm.nih.gov/; accessed on 20 August 2022).

Based on the reviewed studies, several glucosinolate compounds are already isolated from mustard seeds. These include gluconapin, glucoraphanin, glucobrassicin, sinigrin, and sinalbin, to name a few (Figure 2). The major glucosinolates extracted from mustard seeds are sinigrin and sinalbin. As seen in Table 1, sinigrin is particularly abundant in *Brassica juncea*, while sinalbin is the chief glucosinolate in *Sinapis alba*. Sinigrin is responsible for the pungent taste of mustard once it is degraded by myrosinase, while sinalbin has a weaker pungent taste. The level of glucosinolate compound extracted depends on the plant part utilized. Based on the studies reviewed, the seed produces an ample amount of glucosinolate compared to other parts such as the leaves, stalk, and flower [11]. Moreover, some studies tried to investigate the different combinations of mustard species through genetic modification to increase the amount of glucosinolate content, which is not included in this review.

Aside from GSLs, omega-3 fatty acids are also present in the seeds of mustard species (Table 1). Several studies isolated linolenic acid in a significant amount (16.05% of the total fatty acids) from the seeds of *B. juncea* [12,18]. Another study also reported the transgenic production of eicosapentaenoic acid (EPA) in *B. juncea* seed. EPA levels were up to 15% of total seed fatty acids [55]. These studies show that mustard seeds can potentially be tapped as natural sources of GSLs and omega-3 fatty acids. These plants can also be genetically engineered to increase their natural production of the compounds, thus providing a wide array of biological applications.

## 3. Major Extraction Procedures

**Figure 3:**
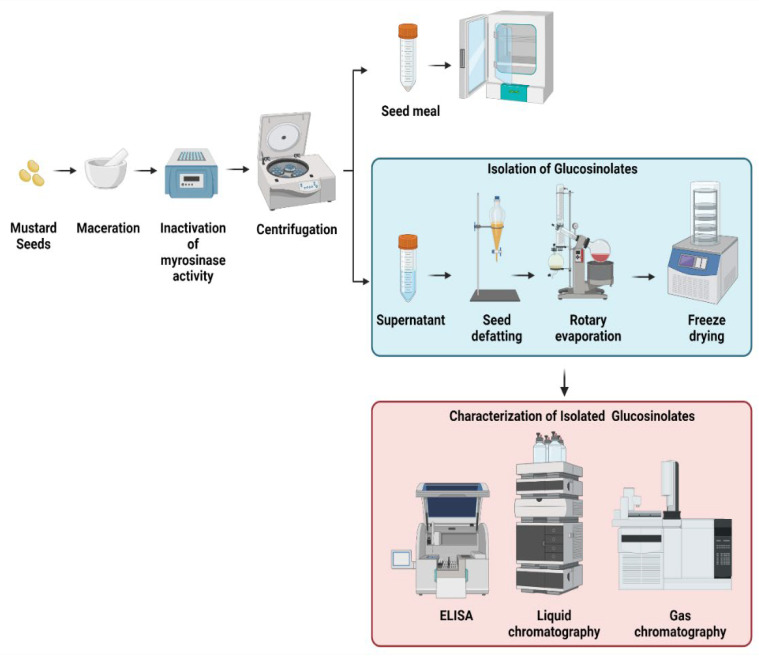
Overview of the extraction, isolation, purification, and structure elucidation of glucosinolates from the seeds of mustard plants. Created with BioRender.com.

In the extraction process of the different mustard seeds, a general procedure is observed (Figure 3). Samples from different mustard species are collected. Some studies utilized either the leaves, stalks, seedpods, or the seed of mustard to determine the glucosinolate content and to determine the fatty acid profile [11,12,20,28,29,40,56]. Generally, the samples are ground and crushed using a mortar and pestle. In some instances, maceration of the seeds is carried out with liquid nitrogen to prevent the conversion of glucosinolate into isothiocyanate. The extraction procedure is usually performed in triplicates by adding a heated or boiling polar solvent, either aqueous methanol or water. This is to inactivate the myrosinase activity. It is because myrosinase is an enzyme that catalyzes the conversion of glucosinolate into isothiocyanate [12,13]. Afterward, the extracted sample is transferred into a conical tube which is then vortexed, centrifuged, and filtered. The supernatant and the seed meal are separated. To further remove the excess solvent, extracts either undergo a rotary evaporator to remove excess methanol or lyophilized to remove excess water. Some studies used seed defatting before analysis of glucosinolate. Seed defatting is done to remove oil and other lipids in the seed. Diethyl ether or petroleum ether is usually used by employing a Soxhlet apparatus. Afterward, an aliquot of the extracts is used for the analysis of GSLs using HPLC, GC-MS, or ELISA via tetrachloropalladate solution, while GC-MS is mainly employed for omega-3 fatty acid profile. 

High-performance liquid chromatography (HPLC) is the standard procedure to separate and quantify glucosinolate in mustard seed [13]. In doing this, an aliquot of the sample is used and is loaded onto a DEAE Sephadex column. Afterward, it is separated using a reversed-phase C-18 column and detected using a photodiode array detector. GSLs are identified and quantified by their characteristics of being aliphatic, benzenic, and indolic [14].

On the other hand, a coupled GC with mass spectrophotometry (GC-MS) is also used in identifying the glucosinolate and omega-3 fatty acid content of the mustard seed [11]. It is because it is suitable for qualitative and quantitative analysis of volatile and semivolatile compounds [12]. It is performed by using a gas chromatograph device and mass spectrometer under a programmed setting. The spectrum of the unknown component is compared to the standard [12,33,35,54,57]. Lastly, enzyme-linked immunosorbent assay (ELISA) is also used to detect glucosinolate, which uses tetrachloropalladate solution. The total glucosinolate is estimated via the complexes that are formed between glucosinolate and the tetrachloropalladate solution. The change in the color produced is measured by the ELISA reader [18,58,59]. Currently, research advances have evolved. Different studies are discovering more ways to determine and to characterize glucosinolate and its fatty acid component in a faster and more efficient way. These include the use of near-infrared spectroscopy.

## 4. Clinical Studies on Glucosinolates and Omega-3 Fatty Acids

### 4.1. Glucosinolates

Although mustard was traditionally used in the medicine of Asian countries, only a few studies were conducted in humans to confirm the bioactivities of its seed and its main compounds. To recognize the beneficial effects of mustard seed extracts and oil action, MEDLINE^®^ and Cochrane Collaboration Central Register of Clinical Trials databases were searched; a summary of the health benefits of GSLs and omega-3 fatty acids is presented in Table 2.

Auriculotherapy is traditionally used in Chinese traditional medicine to treat several diseases. Kim [60] evaluated the effects of the application of white mustard seed for 4 weeks, three times a day on auricular acupressure points on the obesity index in female college students. A reduction in body weight and body mass index (BMI) was observed in all participants. Successively, Kang et al. [61] evaluated the application of white mustard seed on Meridian points on sleep and fatigue in patients undergoing chemotherapy for breast neoplasms. Results of the observational study evidenced that mustard seed application was able to reduce the level of fatigue and improve the physical and psychological conditions of participants. The positive effect of auriculotherapy with mustard seed application was confirmed by Iunes et al. [62]. Forty-four students with temporomandibular disorders and high levels of anxiety were enrolled. The subjects were divided into two groups: an auriculotherapy group and a sham group. The mustard seeds were applied to the sympathetic, brain stem, shenmen, rim, and temporomandibular points. Auriculotherapy associated with mustard seed application significantly reduced the status of anxiety and a decrease in tender points in the submandibular and mandibular regions. A reduction in temporal muscle contraction was also observed.  

More recently, Cândido Dos Reis et al. [63] utilized a similar protocol to evaluate the effect on sleep disorders, anxiety, and the painful symptomatology of temporomandibular disorders. Patients between the ages of 20 and 45 years were enrolled and subjected to the treatment once a week for 8 weeks. A statistically significant reduction in sleep disorder symptoms was observed after the intervention. However, no significant difference was observed for painful temporomandibular disorders and anxiety symptoms.

Goetz et al. [64] reported the effect of mustard seed powder as a possible strategy to improve symptoms of respiratory tract infections. One hundred three participants were enrolled. The treatment consisted of footbaths with powdered mustard seeds once a day for six days. The “Herdecke Warmth Perception” (HeWEF) questionnaire was used to measure the effect of the treatment. Participants in the intervention group showed improvement of “sensation of cold”, “exhilaration,” “unwellness”, and “devotion”. 

Previously, Tian et al. [65] studied the effect of chewing gum with allyl isothiocyanate, a constituent of mustard seed extract, alone and in association with zinc salts on the decrease in oral malodor. Fifteen subjects (aged 20 to 50 years) were asked to chew the trial gum for 12 minutes and the results were compared to a placebo gum. The GC analysis of their breath showed that chewing gum containing allyl isothiocyanate + zinc salts decreased the amount of volatile sulfur compounds (−89% at 1 hour after chewing ended). 

Lett et al. [66] assessed the effects on glycaemic response and satiety of patients after the addition of yellow mustard bran in a potato and leek soup. In this randomized study, 10 healthy, moderately active, and nonsmoking male subjects were recruited. Results revealed the reduction in post-prandial glycaemic response after the addition of yellow mustard bran (5 g) to a soup.

The research on mustard seed oil clinical study evidenced how just one study was completed and its results are published. Summers et al. [67] reported results of a randomized controlled trial that included 500 neonates assigned to full body massage with mustard seed oil. Neonates’ skin integrity was measured over 28 days for parameters including dryness, erythema, rash, pH, stratum corneum cohesion/protein concentration, and trans epidermal water loss. Decreased skin pH was observed in the first week of life. Dryness, erythema, and rash increased during days 1–14 and then decreased by day 28. The trans epidermal water loss increased over time. The gestational age did not modify the effects of the mustard oil. 

### 4.2. Omega 3 Fatty Acids

α-Linolenic acid (ALA) is the main abundant fatty acid of mustard seed oil. Bork et al. [68] studied associations between ALA dietary consumption and the risk of developing ischemic stroke. This Danish Diet, Cancer, and Health study involved 57,053 subjects whose ALA intake was analyzed by using a validated semiquantitative food frequency questionnaire. A total of 1859 ischemic strokes were recorded in four years of observation; however, multivariable analyses did not reveal any type of association between ALA intake and the incidence of ischemic stroke regardless of stroke subtypes.

Previously, Hennebelle et al. [69] examined the effects of an ALA-rich supplement on plasma long-chain n-3 polyunsaturated fatty acid PUFAs and ketogenic response. Results evidenced that the supplement slightly stimulated post-prandial ketogenesis. The effect of ALA on diabetes type 2 (T2DM) patients was assessed by several clinical studies; however, Jovanoski et al. [70] who conducted a systematic review and meta-analysis, concluded that diets rich in ALA did not influence parameters altered in T2DM, such as fasting blood glucose and insulin and glycated hemoglobin. 

Recently, Bjornevik et al. [71] investigated the association between ALA levels and severity of multiple sclerosis (MS) in 87 patients. Results showed that ALA supplementation is a good strategy to counteract the severity of this disease. Recently, Burak et al. [72] investigated the combined effect of ALA (3.6 g/day) and quercetin (190 mg/day) administration for 8 weeks on antioxidant status, blood pressure, lipid and glucose metabolism, and biomarkers of inflammation in healthy patients. At the end of the study, data from 67 individuals with a mean age of 24.6 years were recorded. The association ALA + quercetin reduced total cholesterol, apolipoprotein B, and low-density lipoprotein cholesterol by a statistically considerable amount. However, no significant evidence was found on markers of cardiovascular disease risk, including the effect on blood pressure. This evidence was successively confirmed by Pieters et al. [73]. 

Some clinical studies investigated the effect of ALA in obese patients. Saito et al. [74] assessed the effects of ALA-rich triacylglycerol (ALA-TAG) and ALA-rich diacylglycerol (ALA-DAG) diet on the visceral fat area in obese patients. One hundred patients, divided into two groups, were invited to consume for twenty weeks 2.5 g/day ALA-DAG or ALA-TAG. At the end of the observational period, the BMI and visceral fat area were suggestively reduced by the ALA-DAG treatment. Moreover, ALA-DAG remarkably decreased the baseline of the fasting TAG serum concentration. 

The effect of the association ALA-DAG on dietary fat oxidation in comparison with control TAG alone was assessed by Ando et al. [75]. In this intervention trial, 16 subjects were invited to consume either 2.5 g/day ALA-DAG or TAG for 14 days, separated by a 21-day washout period. Additionally, in this case, it was possible to show that ALA-DAG treatment significantly enhanced fat utilization. Successively, Egert et al. [76] analyzed and compared the effect of an energy-restricted diet on fatty acids composition of serum phospholipids in patients with metabolic syndrome. For this purpose, 81 obese or overweight patients with features of metabolic syndrome were enrolled. At the end of 26 weeks of treatment, the authors highlighted that the participants treated with a low-calorie diet high in ALA did not show alterations in the picture of serum phospholipids and did not show an increase that led to higher concentration of eicosapentaenoic acid.

GSLs are ingested in an inactive form and successively, when vegetables are cut or chewed, are converted into some degradation products such as thiocyanates, isothiocyanates, etc., by the enzyme myrosinase. They are reported to be present generally in the Brassicaceae family [78]. These compounds are particularly abundant in yellow (*Sinapis alba*) and Indian or brown (*Brassica juncea*) mustard seeds, although with qualitative and quantitative differences [17]. In fact, sinigrin, glucoiberin, epiprogoitrin, gluconapin, gluconasturtiin, and gluconeobrassicin are the main abundant compounds in *B. juncea*, whereas sinalbin and glucoraphanin are found in *S. alba*. After several epidemiological studies, it is possible to assert that the consumption of cruciferous-rich diets leads a series of beneficial effects on human health. These effects are attributed to GSLs and their breakdown products, isothiocyanates. Among them, sulforaphane, derived by the hydrolysis product of glucoraphanin, has been reported to have several beneficial effects. 

Recently, researchers discovered that mustard seeds contain a more resistant form of myrosinase, which is why adding mustard to broccoli increases the formation of sulforaphane. This compound can inhibit *Salmonella* and *E. coli* growth in the small intestine. To demonstrate the synergy between broccoli and mustard, Okunade et al. [77] measured the urinary concentration of sulforaphane *N*-acetyl-l-cysteine, a metabolite of sulforaphane in twelve healthy adults after ingesting cooked broccoli (200 g), with and without brown mustard powder (1 g). The results showed the addition of mustard increases the bioavailability of sulforaphane by more than four times as the *N*-acetyl-l-cysteine sulforaphane excreted was 9.8 versus 44.7 μmol per g of creatinine for participants who consumed cooked broccoli alone and in association with powdered mustard seeds, respectively.

## 5. Pharmacological Potential of Glucosinolates and Omega-3 Fatty Acids

Mustard seeds are characterized by the presence of secondary metabolites [79,80] mainly including phenolic compounds, GSLs, and omega-3 PUFAs that have attracted the attention of numerous researchers. Herein, we report their pharmacological potential highlighting the mechanisms of action.

### 5.1. Glucosinolates

GSLs are converted into several products of degradation including thiocyanates and isothiocyanates (ITCs). These molecules are demonstrated to possess different biological properties including protection against pathogens and anticarcinogenic effects by their ability to inhibit the formation of exogenous or endogenous carcinogens. As reported in Table 3, in vivo studies showed that several GSLs hydrolysis products, in particular ITCs, have cytotoxic activity against different cancer cells and protective properties against chemical-carcinogen-induced cancer [81,82,83,84,85,86,87,88]. Rose et al. [89] investigated the ability of 4-methysulfinylbutyl and 7-methylsulphinylheptyl ITCs, extracted from *Rorippa nasturtium-aquaticum* and *Brassica oleracea*, to suppress in vitro the potential invasivity of the MDA-MB-231 tumor cell line and to inhibit metalloproteinase 9. 

Several studies showed that sulforaphane (SFN) is one of the most promising anticancer agents. SFN inhibited PC-3 (human prostate) cancer cells proliferation by inducing apoptosis and also prevented the mammary tumorigenesis induced by 9,10-dimethyl-1,2-benzanthracene [102,108]. Moreover, in rats, SFN considerably inhibited the formation of azoxymethane-induced colonic aberrant crypt foci formation in rats The proapoptotic activity of SNF can be attributed to its ability to downregulate Bcl-2, activate caspases-3, -8, and -9, and upregulate Bax [102]. 

Kan et al. [122] showed that SFN inhibited several cancers-associated signaling pathways, such as P53, phosphorylated nuclear factor-κB, caspase-3, phosphorylated AKT, B-cell lymphoma 2 (Bcl-2), P27, Bcl-2-associated X protein, cMyc, Cyclin-D 1, and cytochrome c, and decreased the levels of expression of epidermal growth factor receptor 2 (HER2) in the human ovarian cancer cell line. Interestingly, SFN acted in synergism with cisplatin to enhance apoptosis and inhibit cancer cell proliferation. The ability of SFN to suppress cancer growth was confirmed by xenograft experiments in vivo [122].

Recently, the effects of ITCs (SFN and PEITC) on DNA damage and replication in PC-3 tumor cells, prostate epithelial cells (PNT2), and normal fibroblasts (HDFa) were analyzed [123]. Both SFN and PEITC inhibited the replication of DNA, followed by double-strand breaks (DSB), which were more marked in PC-3 cells. The selective antiproliferative effects demonstrated by SFN and PEITC toward investigated tumor cell lines derived from less effective DNA repair in these cell lines in comparison to the normally used cell lines. 

The inhibition of these enzymes promotes cells protection against DNA damage produced by different carcinogens and reactive oxygen species. The nuclear factor erythroid 2 related factors 2/antioxidant response element pathway is the main determinant of the gene induction of enzymes of phase II. Among GSL hydrolysis products, ITCs are shown to be strong inducers of phase II enzymes activity by increasing the transcription of genes that contain ARE [124]. SFN is an active inducer of enzymes of phase II. This ITC showed indirect antioxidant activity probably related to the induction of quinine reductase, heme-oxygenase, and glutathione transferases [125]. 

ITCs not only exhibited antioxidant activity through the upregulation of ARE-driven genes, but also demonstrated to be potent activators of NrF2 and to decrease the inflammatory responses *via* the NFκB pathway [126]. PEITC and 8-methylsulfinyloctyl isothiocyanate (MSO) were examined for their potential ability to modulate the inflammatory response in lipopolysaccharide (LPS)-induced RAW 264.7 macrophages by assessment of cyclooxygenase-2 (COX-2) and inducible nitric oxide synthase (iNOS) expression [118]. Both PEITC and MSO iNOS protein and COX-2 expression levels are in association with the inactivation of NFκB. As demonstrated for other ITCs, Boyanapalli et al. [127] have shown that the anti-inflammatory activity of PEITC is also linked to its interaction with the NrF2 pathway.

Several studies showed that the ITC erucin induced HO-1 expression through p38 signaling and NrF2 via ERK1/2, p38-MAPK, and JNK pathways [128]. Previously, Yehuda et al. [129] also reported the ability of erucin to decrease the transcription of proinflammatory agents, such as TNF-α, IL-1, and IL-12, in THP-1 cells treated with LPS. Moreover, erucin also demonstrated its remarkable anti-inflammatory effects in LPS-stimulated macrophages through the inhibition of NFκB signaling [130].

Numerous works concerning the antimicrobial activity of GSLs are present in the literature and several studies are evidenced as in part responsible for the antimicrobial properties of GSLs and their hydrolysis products. In this regard, some mechanisms are proposed. Among them, Kojima [131] proposed that these compounds can determine the obstruction of the synthesis of ATP in bacteria by the uncoupler action of oxidative phosphorylation in mitochondria. Moreover, GSL hydrolysis products can act by inactivating several bacteria enzymes through the oxidative breakdown of the S–S– bridges [132].

Borges et al. [133] investigated the antibacterial activity of 2-phenylethylisothiocyanate (PEITC) and allylisothiocyanate (AITC) against *Staphylococcus aureus*, *Listeria monocytogenes*, *Escherichia coli*, and *Pseudomonas aeruginosa*, finding an MIC (minimal inhibitory concentration) of 100 μg /mL against all tested bacteria. These results agree with those reported in other works. Pang et al. [134] demonstrated that AITC possesses antimicrobial effects against *P. aeruginosa* (ATCC 10145, 15442, and 27853), extending the shelf-life of catfish fillets. AITC exhibited MIC values of 50, 100, and 200 µg/mL against *E. coli*, *L. monocytogenes*, and *S. aureus*, respectively [135]. Successively, Luciano and Holley [136] revealed MIC in the range 25.5–510 µg/mL with the raising of pH for AITC against *E. coli* O157:H7. 

Conrad et al. [137] studied a mixture of AITC, PEITC, and benzyl-ITC against *E. coli*, *P. aeruginosa*, *Haemophilus influenzae*, *Klebsiella pneumoniae*, *Moraxella catarrhalis*, *Proteus vulgaris*, *S. aureus*, *Serratia marcescens*, *S. pyogenes*, and *Streptococcus pneumoniae*. For Gram-positive bacteria, the ITCs MBC (minimum bactericidal concentration) was > 1000 µg/mL. The same results were found for PEITC against *P. aeruginosa* and *E. coli*. Considering the MIC and MBC results, AITC and PEITC may be considered as nonspecific antimicrobial agents on both Gram-positive and Gram-negative bacteria. Indeed, the presence, along with the cytoplasmic membrane, of an outer membrane in Gram-negative bacteria, did not increase the antimicrobial resistance of *P. aeruginosa* and *E. coli*.

*Helicobacter pylori* infection increases the risk for developing gastric cancer. The hydrolytic product of glucoraphanin, namely sulforaphane (SFN), demonstrated potent bacteriostatic effects against three standard strains and 45 clinical isolates of *H. pylori*. Additionally, short-term exposure to SFN removed *H. pylori* from the Hep-2 cell line. In another work, the administration of SFN for five days eliminated *H. pylori* from eight out of eleven xenografts of human gastric tissue implanted in immunocompromised mice [138]. Aires et al. also showed the potential antibacterial activity of GSLs and their hydrolysis products against bacteria isolated from the human intestinal tract [139]. In this work, the most effective GSLs hydrolysis products were ITCs with benzyl-ITC and sulforaphane as the most active growth inhibitors. Indole-3-acetonitrile showed inhibitory activity against Gram-negative bacteria, while indole-3-carbinol exhibited inhibitory activity against Gram-positive microorganisms but not against Gram-negative bacteria. 

Generally, ITCs are more effective than other GSLs hydrolysis products, and aromatic ITCs are more active compared to aliphatic ITCs. ITC can react nonenzymatically with amino and thiol groups to form thioureas and dithiocarbamates, respectively, compounds that can contribute to the antibacterial properties of ITCs by inhibiting enzymes and/or essential proteins and increasing oxidation, consequently leading to bacterial cell death [124,140]. Moreover, ITC, interacting with cytochrome P-450 enzymes, can be oxidized and produce ITCs more reactive than the parent compounds [141].

Mustard oils show inhibitory activity against fungi [142,143,144]. This activity may be related to the presence of allyl and phenethyl ITCs, although, of course, each compound shows a specific activity and the activity ranges differ with changes in the ITCs substituent groups [142]. Kojima [131], using three different *Saccharomyces cerevisiae* strains, described the ability of ITCs to stop coupling between reactions of phosphorylation and electron transport, consequently blocking ATP synthesis. Studies have demonstrated fungicidal activity of 2-propenyl-ITC against pear *P. expansum*; allyl-ITC against strawberry *Botrytis cinerea*, nectarine and peach *Monilinia laxa*, and pizza crust *Aspergillus parasiticus*; and benzyl-ITC against tomato *A. alternata*, cotton *Phymatotrichopsis omnivora*, and grapes, soybeans, green coffee, and peanuts *Aspergillus ochraceus* [145,146,147,148,149,150,151].

Recently, Zhang et al. [152] evaluated the antifungal activity against *A. alternata* of 2-phenylethyl-ITC (2-PEITC) in pear fruit; 2-PEITC remarkably inhibited *A. alternata* spore germination and mycelial growth and significantly decreased the expansion of black spot rot on pears that had been treated with *A. alternata*. Choi et al. [153] assessed the antifungal activity ITCs to find natural antifungal agents against pathogenic dermal fungi. ITCs inhibited the growth of *Epidermophyton floccosum*, *Trichophyton mentagrophytes*, *T. rubrum*, and *Microsporum canis* pathogenic dermal fungi with minimum fungicidal concentrations of 200 µg/mL. 

### 5.2. Omega-3 Fatty Acids

In recent decades, research on *n*-3 PUFAs has grown exponentially. In fact, *n*-3 PUFAs have been shown to play a critical role in neuronal cell function and in immune and inflammatory reactions, and many studies have revealed the benefits of *n*-3 PUFAs in diabetes mellitus, obesity, cardiovascular disease, atherosclerosis, dyslipidemia, metabolic syndrome, hypertension, neurological/neuropsychiatric disorders, osteoporosis, and renal diseases [5,154]. 

Several review articles have reported the existing knowledge on the chemistry, bioavailability, dietary sources, potential deficiency states, and biological properties of *n-*3 PUFAs [5,10,154,155,156,157,158,159]. Recently, Oppedisano et al. [159] described the antioxidant and anti-inflammatory properties of *n*-3 PUFAs and their role in preventing and/or treating cardiovascular diseases. In fact, several research reports have noted the ability of *n*-3 PUFAs to decrease endothelial cell apoptosis and oxidative stress-related mitochondrial dysfunction through the increased activity of endogenous antioxidant enzymes, and to counteract the release of proinflammatory cytokines in the myocardium and vascular tissues, thus restoring the activity of the myocardium and the integrity of vascular tissues. However, further studies involving large numbers of patients are necessary to confirm their potential use to reduce and/or to treat cardiovascular diseases.

McGlory et al. [158] analyzed available literature data on the potential enhancement of skeletal muscle anabolism by *n-*3 PUFAs intake. An increase in strength and muscle mass in healthy older people following supplementation with *n*-3 PUFA was also observed in subjects who experienced a loss of muscle mass due to prolonged immobility. EPA and DHA incorporation into membrane phospholipids is found as the principal means by which *n*-3 PUFAs positively impact skeletal muscle. 

The incorporation of these *n-*3 PUFAs into membrane phospholipids has been proven to lead to a reduction in the expression of some factors that regulate muscle protein breakdown, the enhancement of mitochondrial respiration kinetics, and the rate of synthesis of muscle proteins. However, how EPA and DHA incorporation into membrane phospholipids can modify these processes is not yet known. Of considerable interest is the potential for *n*-3 PUFAs to counteract the atrophy of muscles and to stimulate recovery from periods of muscle disuse. Studies have been carried out, but much additional research must be performed before drawing conclusions concerning the effectiveness of *n*-3 PUFAs intake on musculoskeletal health. Some important questions to be answered concern, in particular, the possibility to discern, given their independent biological actions, the independent role of EPA and DHA in producing modifications in skeletal muscle plasticity. Another factor of interest is the evaluation of potential off-target effects of increased intake of *n*-3 PUFAs and whether there are negative consequences in other vital processes.

Avallone et al. [160] critically described clinical trials and epidemiological studies that evaluated the impact on neurodegenerative disorders, mainly on Alzheimer’s disease (AD) and Parkinson’s disease (PD), by dietary intake of *n*-3 PUFAs that represent potentially interesting agents for the treatment of these diseases. Two important studies, namely, “Nurses’ Health Study” (1984–2000) and “Health Professionals Follow-Up Study” (1986–2002), analyzed the association between the potential risk of PD and dietary lifestyle. Two dietary styles, namely the prudent diet (high consumption of fish, vegetables, and fruit) and the Western diet (high consumption of red meats, refined grains, and high-fat dairy products) have been recognized and compared. The prudent diet was demonstrated to significantly reduce the risk of PD, while the Western diet did not [161]. Additionally, in The Rotterdam Study, PUFAs consumption was related to a lower PD risk [162]. Data obtained in The Rotterdam Study, successively confirmed by other studies, revealed that the consumption of *n*-3 PUFAs, oils rich in *n*-3 PUFAs, fatty fish, or a diet with high consumption of fish, vegetables, and fruit, is connected to a reduction in the potential risk of occurrence of AD [163,164,165]. 

The protective activity against AD by *n*-3 PUFAs was investigated in the RBMVECs (rat brain microvascular endothelial) cell line [166]. This study demonstrated that the activity of catalase, superoxide dismutase, and glutathione peroxidase was improved, and ROS and lipid peroxidation was reduced after incubation of cells with *n*-3 PUFAs. A reduction in the amount of apoptotic RBMVECs was also described.

Most of the studies have focused on investigating mixtures of *n*-3 PUFAs and not individual fatty acids. In recent years, evidence for the effects of DHA, DPA, and EPA has grown. For example, with neurodegenerative diseases, such as AD, the focus of several studies has been on DHA because of its essential role in the growth and functional development of the brain. Different effects, including the modulation of key properties such as membrane fluidity, permeability, compression, fusion, and protein activity, have been described for DHA [167,168,169]. 

Increasing the phosphatidylserine levels of neuronal membranes may affect neuronal survival through the phosphoinositide 3-kinase/serine/threonine-protein kinase pathway [170]. DHA exerts an important role in modulating phosphatidylserine synthesis [171,172]. The dietary intake of EPA and DHA similarly improved the levels of brain phospholipids [173].

## 6. Conclusions and Future Perspectives

In summary, this review shows the different GSLs and omega-3 fatty acids from mustard seeds, extraction procedures from mustard seeds, and preclinical and clinical studies supporting the use of these compounds in improving human health. Previous in vitro, animal, and human studies showed that these compounds may be further developed as potential treatments for infections, cancer, diabetes, and metabolic syndrome. However, further studies are still needed to investigate action mechanisms of these naturally occurring GSLs and omega-3 fatty acids, together with their safety and efficacy. Since these compounds are being developed for pharmacologic use in humans, there is also a need to improve and standardize the extraction, isolation, and characterization methods for GSLs and omega-3 fatty acids from mustard seeds. This will also be useful in the quality control of these compounds for large-scale production and commercialization.

## Figures and Tables

**Table 1:** List of major glucosinolates and omega-3 fatty acids in each mustard seed species and its separation technique.

Glucosinolate Compound *	Plant Material	Isolation Technique	Reference
***Brassica juncea***			
(1)	Seed	GC-MS	[12]
(2)	Seed	HPLC	[13]
(2)	Seed meal	HPLC	[14]
(2)	Seed	HPLC DART-MS	[15]
(3)	Seed	RP-UHPLC-PDA-ESI-MSn	[16]
(2) (4)	Seed	Process optimization and innovative pretreatment (high voltage electrical discharges)	[17]
(1)	Seed meal	ELISA at 405 nm (tetrachloropalladate solution)	[18]
(2)	Seed	HPLC	[19]
(2)	Roots and stubble, straw, seed	HPLC	[20]
(2)	Seed meal	HPLC-MS	[21]
(2)	Seed meal	HPLC	[22]
(2); (4)	Seed	HPLC	[23]
(2)	Seed	HPLC-TOF-MS	[24]
(2)	Stem Leaves	HPLC	[25]
(2)	Seed Seed meal	HPLC/UV	[26]
(2)	Seed	HPLC/UV Ion chromatography HPLC/MS	[27]
(2); (4); (5); (6); (7); (8); (9)	Seed Stalk	HPLC-MS	[28]
(2)	Leaves Seed meal	HPLC HPLC-MS	[29]
(2)	Seed meal	HPLC	[30]
(2)	Seed	HPLC	[31]
(2)	Seed	HPLC	[32]
(10)	Seed meal	GC	[33]
(2); (4); (11)	Seed	Near-infrared spectroscopy	[34]
(2); (4); (6); (12)	Seed	GC	[35]
(3)	Seed	HPLC	[36]
(2); (4); (6); (13); (14)	Seed	GC	[37]
(2); (16)	Seed	Ion-pair HPLC	[38]
(2); (16)	Seed	HPLC	[39]
(1)	Seed Leaves	HPLC	[40]
(2)	Seed	HPLC	[41]
(2); (4); (6); (9); (11); (15)	Seed	NIRS HPLC	[42]
(2); (6)	Flowers, seed pods, seeds, leaves, stems, stalks, roots	HPLC	[11]
(2); (4); (13)	Seed	HPLC	[43]
***Sinapis alba***			
(2)	Seed meal	HPLC	[14]
(3)	Seed	HPLC DART-MS	[15]
(3)	Seed	RP-UHPLC-PDA-ESI-MSn	[16]
(2); (4); (5); (7); (8); (9); (11); (16); (17); (18); (19); (20)	Seed	HPLC-PDA-ESI-MSn	[44]
(3)	Roots and stubble, straw, seed	HPLC	[20]
(2); (3)	Seed meal	HPLC	[22]
(3)	Seed	HPLC-TOF-MS	[24]
(3)	Seed Seed meal	HPLC/UV	[26]
(3)	Seed	HPLC/UV; Ion chromatography; HPLC/MS	[27]
(10); (21)	Seed meal	GC	[33]
(2); (4); (6); (12)	Seed	GC	[35]
(2); (3)	Seed	HPLC	[45]
(3)	Seed	HPLC	[46]
(3); (16)	Seed	Strong ion-exchange displacement centrifugal partition chromatography (SIX-CPC) HPLC	[47]
(2)	Seed	HPLC	[41]
(3)	Seed	Ion-exchange centrifugal partition chromatography	[48]
***Brassica nigra***			
(2); (4); (8); (9); (11); (13); (16); (17); (19); (22)	Seed meal	HPLC	[49]
(2); (3)	Seed	HPLC DART-MS	[15]
(2); (4); (6); (12)	Seed	GC	[35]
(2); (16)	Seed	Ion-pair HPLC	[38]
(2); (4); (6); (9); (11); (15)	Seed	NIRS HPLC	[42]
***Brassica carinata***			
(2); (4); (8); (11); (13); (16); (17); (19); (22)	Seed meal	HPLC	[49]
(2); (3)	Seed meal	HPLC	[22]
(4); (10)	Seed	Fourier transform infrared spectroscopy	[50]
(23)	Seed	HPLC	[51]
(2); (4); (6)	Seed	HPLC	[52]
(2)	Seed	HPLC	[46]
(2); (4); (6); (9); (11)	Seed	NIRS HPLC	[42]
***Brassica elongata***			
(6)	Seed	LC-MS	[53]
(24)	Seed	GC	[54]
(2); (4); (6); (9); (11); (15)	Seed	NIRS HPLC	[42]
***Brassica hirta***			
(2); (3); (6)	Flowers, seed pods, seeds, leaves, stems, stalks, roots	HPLC	[11]

* Refer to Figure 2 for the structure and name of the glucosinolates and omega-3 fatty acids.

**Table 2:** Importance of glucosinolates and omega-3 fatty acids with respect to health benefits.

Mustard Seed/Compound Source	Biological Activity	References
White mustard seed	Auriculotherapy Reduces body weight and body mass index	[60]
White mustard seed	Reduces fatigue Improves the physical and psychological condition	[61]
White mustard seed	Auriculotherapy Reduces anxiety and temporomandibular muscle contraction	[62,63]
Mustard seed powder	Improves respiratory tract infections	[64]
Mustard seed extract/Allyl isothiocyanate	Reduces volatile sulfur compound causing oral malodor	[65]
Yellow mustard bran	Reduces postprandial glycemic response	[66]
Mustard seed oil	Effect on the epidermal integrity	[67]
Mustard seed oil/α-Linolenic acid (ALA)	Association of ALA intake and ischemic stroke	[68]
ALA	Stimulates postprandial ketogenesis	[69]
ALA	No effect in fasting blood glucose and insulin and glycated hemoglobin	[70]
ALA	Reduces the severity of multiple sclerosis	[71]
ALA + quercetin	Decreases total cholesterol, LDL, apolipoprotein B	[72,73]
ALA-rich triacylglycerol (ALA-TAG) ALA-rich diacylglycerol (ALA-DAG)	Reduction in BMI and visceral fat with ALA-DAG	[74]
ALA-rich diacylglycerol (ALA-DAG)	Enhances fat utilization	[75]
ALA	Effect of ALA-rich diet on the fatty-acid composition of serum phospholipids in obese patients affected by metabolic syndrome	[76]
*Sinapis alba* (yellow mustard)/Glucoraphanin	Inhibits *Salmonella* and *E. coli* growth	[77]

**Table 3:** Antiproliferative activity of GSLs hydrolyzed compounds (isothiocyanates, ITCs).

Compounds	Cell Lines/In Vivo Models	Activity	Reference
Benzyl-ITCs	HT29 colon carcinoma cells	Apoptosis induction	[90]
	BxPC-3 cells	Cell cycle arrest, apoptotic induction, inhibition of NF-κB	[91]
	Hamsters	Protection against pancreatic carcinogenesis initiation	[92]
	Caco-2 and LS-174 cells	Growth inhibition	[93]
	HNSCC head and neck squamous cell carcinoma cell line	Activation of PARP cleavage and caspase-3	[94]
Allyl-ITCs	Swiss albino mice	Inhibition of cyclophophamide-induced urotoxicity	[95]
	PC-3 xenografts	Growth inhibition	[96]
	LNCaP cells	Apoptosis induction and growth inhibition by G2/M arrest	[97]
	Human myeloblastic leukemia-1 cells	Inhibition of HL60 (p53-) and (p53þ)	[98]
4-Methylsulfinylbutyl-ITCs	Hamsters	Protective activity against pancreatic carcinogenesis initiation	[92]
	MDA-MB-231 cells	Growth inhibition	[89]
	Mice	Benzo(a)pyrene-induced forestomach cancer inhibition	[99]
	L-1210 and ME-18 cells	Growth inhibition and induction of apoptosis	[100]
	HepG2 cells	Growth inhibition	[101]
	PC-3 cells	Caspases-mediated apoptosis	[102]
	Medulloblastoma cells	Caspases-mediated apoptosis	[103]
	DU-145 cells	Growth inhibition	[104]
	LNCaP cells	Growth inhibition	[93]
	Human T-cell leukemia	Induction of apoptosis and cell cycle arrest	[105]
	HT29 cells	Growth inhibition	[106]
	F344 rats	Azoxymethane-induced colonic crypt foci inhibition	[107]
Phenylethyl-ITCs	F344 rats	Tumorigenicity and 4-(methylnitrosamino)-1-(3-pyridyl)-1-butanone-induced DNA adduct inhibition	[84]
	Rats	Azoxymethane-induced colonic crypt foci inhibition	[108]
	DU-145 and LNCaP cells	Enhancement of p21 protein and G0–G1 arrest	[109]
	F344 rats	Azoxymethane-induced colonic crypt foci inhibition	[107]
	p53-deficient PC-3 cells	Apoptosis induction	[110]
	LNCaP cells	Apoptosis induction	[111]
	Rats	Urinary bladder tumorigenesis inhibition	[112]
	HT29 cells	Caspase-3 activation and Inhibition of NF-κB activity	[113]
	HL60 cells	Protein kinase C inhibition	[114]
	Leukemia and human bladder carcinoma cells	Growth inhibition	[115]
	Rats	4-(Methylnitrosamino)-1(3-pyridyl)-1-butone-induced pulmonary neoplasia	[116]
	Ovarian cancer cells	Apoptosis induction	[117]
7-Methylsulfinylheptyl-ITCs	MDA-MB-231 cells	Suppression of activity	[118]
Indole ethyl-ITCs	SH-S454, SMS-KCNR, SK-N-SH, IMR-32 cells	Anti-proliferative and apoptotic effects	[119]
Phenylmethyl-ITCs	HeLa cells	Caspase-3 activation	[120]
Phenyl-ITCs	Swiss albino mice	Cyclophophamide-induced urotoxicity inhibition	[95]
Phenylbenzyl-ITCs	HeLa cells	Caspase-3 activation	[120]

Some portions of the table are reproduced from Vig et al. [121] with permission (originally Table 6).

## Data Availability

All data are presented in the form of tables and figures within the manuscript.
